# Prevalence of burnout among military personnel in the plateau region of China: a cross-sectional survey

**DOI:** 10.1186/s12889-024-19340-w

**Published:** 2024-07-16

**Authors:** Lei Shi, Fei Ren, Shen Xin, Qin Sun, Dan-ni Li, Ke Li, Yuan Wang

**Affiliations:** Department of Medical Research, The Ninth Medical Center, Chinese PLA General Hospital, Beijing, 100101 China

**Keywords:** Burnout, Military Personnel, Plateau Region

## Abstract

**Objectives:**

The geographical environment and military activities in the plateau area pose potential work-related stressors for military personnel, leading to burnout which is an external manifestation of internal energy exhaustion caused by stress. Without countermeasures, this can result in serious military problems. This study aims to examine the association between burnout and occupational stressors among military personnel stationed in the plateau area of China.

**Material and methods:**

A stratified randomized cluster sampling survey was conducted among 2026 military personnel from 6 different troops stationed in the plateau area of China. The Chinese Maslach Burnout Inventory-General Survey(MBI-GS in Chinese) was administered from March 2022 to December 2023, and data were analyzed using SPSS version 25.

**Results:**

A total of 2026 military personnel participated in the survey. The mean overall burnout score was 3.37 ± 0.73, with emotional exhaustion at 2.69 ± 0.89, depersonalization at 3.58 ± 0.92, and professional achievement at 3.81 ± 0.85 levels respectively reported by participants on average scale scores ranging from zero to six. Severe level of burnout was reported by 43.2% of participants while medium level of burnout was reported by 54 .3%. Age, education level, length of military service, and household income were identified as important factors influencing burnout.

**Conclusion:**

This study highlights a relatively high prevalence of burnout among military personnel stationed in plateau areas necessitating attention towards their occupational health particularly focusing on working hours and economic aspects so as to formulate effective policies and implement intervention measures that strengthen career development for soldiers deployed in such regions.

## Background

Burnout is a psychological syndrome characterized by various physical reactions [1]. It arises from prolonged exposure to work-related stressors, resulting in diminished energy and enthusiasm towards one's job [2], as described by Maslach through emotional exhaustion (a loss of power and inability to connect with clients), depersonalization (displaying detached and emotionless behavior towards others while holding negative attitudes), and personal achievement (having a negative perception of one's work efforts and feeling stagnant) [3]. Burnout poses a significant threat to the work ability of employees, particularly those in people-oriented occupational groups [4].

Numerous studies have identified burnout among different populations, including 68.2% of healthcare workers [5], 40% of teachers [6], and 23.4% of police officers [7]. Emerging evidence suggests that burnout, as a maladaptive response to stress, not only increases the risk for depression and anxiety disorders [8–10] but also elevates the likelihood of developing cardiovascular diseases and other physical ailments such as insomnia [11], suicidal tendencies [12], gastric disorders [13], ulcers, headaches [14], and arrhythmias [15]. Similar findings may be observed among military personnel due to the high-pressure nature inherent in their profession.

Military operations are characterized by long working hours, heavy workloads, and a high level of responsibility, all of which may contribute to burnout [16]. A systematic review has revealed that the prevalence of high burnout among soldiers ranges from 0.9% to 40% [17]. A survey conducted by the Brazilian Air Force found that 86.7% of military professionals serving as Air Traffic Controllers experienced burnout [18]. In the US Army, it was discovered that 43.4% of soldiers experienced burnout, with this situation worsening with age [19]. Comparatively, the detection rate of burnout among teachers is lower at 68% for university teachers and 78.5% for middle school teachers; however, in the Cameroonian army, it reaches as high as 85% [20]. Additionally, the military environment often entails significant stress due to life-threatening situations and the need for quick decision-making processes which can negatively impact an individual's mental and physical well-being [21]. Combat-related experiences have been identified as a specific source of substantial stress among US military personnel stationed in Afghanistan, thereby increasing their risk of burnout [22]. Furthermore, the military organizational culture emphasizing discipline and obedience may also contribute to feelings of isolation and lack autonomy among military personnel. The consequences of burnout among military personnel can be profound including decreased job satisfaction, reduced productivity levels, and increased turnover rates [23]. Burnout can also have detrimental effects on both physical and mental health in military personnel leading to symptoms such as insomnia, depression, and anxiety [24, 25]. Moreover, burned-out personnel may exhibit lower motivation levels and diminished ability to perform duties effectively thus impacting overall performance and effectiveness within the entire military organization [20, 26].

In plateau regions, military personnel face unique challenges and pressures that can lead to burnout. The harsh plateau environment, characterized by low oxygen levels, extreme temperatures, intense ultraviolet radiation, and other factors, significantly impacts the physical and mental well-being of military personnel [27]. Alcantara-Zapata's research has revealed that exposure to high altitudes can have an impact on individuals' stress levels [28]. Factors such as low oxygen and low pressure can contribute to negative emotion, as well as a higher degree of depression and anxiety [29]. It has been demonstrated that negative emotional experiences can influence the level of burnout experienced by individuals [30]. Prolonged exposure to these harsh conditions easily results in physical fatigue, mental stress, and reduced job satisfaction which ultimately contribute to burnout [31]. Furthermore, military personnel working in plateau regions often experience high pressure and responsibility [32]. This stems from the fact that in plateau areas, high-intensity training content is a severe challenge to individual tolerance. This is because the exercise capacity of individuals is reduced in a low oxygen environment [33]. They must remain constantly vigilant to handle various emergencies and challenges. For example, there are potential risks to military security in border areas. This demanding work necessitates maintaining a heightened level of mental tension for extended periods of time which can readily lead to stress and burnout. In addition, the link between sleep and stress has also been well established, as sleep disorders have become a source of stress when they begin to have adverse effects [34]. A cross-sectional study of sleep quality and stress in the German army [35] found that soldiers had a higher incidence of sleep problems than the general population, and similar rates were found among U.S. troops in Afghanistan [36]. Taylor [37] found that soldiers with insomnia had more stress, physical and mental problems than those without insomnia. However, altitude exposure can lead to insomnia in individuals, which may be related to the recovery of neural circuits and endocrine function, which also become one of the potential stressors. Although the officer managers have intervened in the health problems of soldiers in plateau areas through physical examination, psychological screening and other ways, the stress-related problems are still prominent [38]. Numerous previous studies have demonstrated that military burnout in plateau areas is associated with various factors. However, the specific challenges faced by Chinese military personnel experiencing burnout in plateau areas have not been thoroughly investigated. Therefore, this cross-sectional study aims to assess the prevalence of burnout among soldiers in plateau areas of China.

## Methods

### Study population

This study is based on a random cross-sectional multi-center survey conducted among military personnel stationed in China's plateau region. A stratified randomized cluster sampling approach was employed to select participants from six different troops. Random numbers ranging from 1 to 50 were generated using computers and assigned internally within all investigation troops' institutions. Based on the classification of different cities within the region, eight camps were located in Xining(3137 m of altitude), four camps in Golmud(3627 m of altitude), twenty-four camps in Lhasa(3650 m of altitude), and four camps in Shigatse(3836 m of altitude). Thirty camps were selected for questionnaire surveys based on proportional representation determined by the size of the random number; this included six camps in Xining, three camps in Golmud, eighteen camps in Lhasa, and three camps in Shigatse.

### Survey sampling

The study was conducted from March 2022 to December 2023. Researchers visited a military site and administered questionnaires to investigate the selection results. A total of 2050 military personnel from 30 camps belonging to 6 troops were selected as research subjects for the questionnaire survey, with a response rate of 99.2% (2034/2050). Two researchers, both trained in psychology, provided guidance and explanations while informing participants about the purpose and methodology of the study. Participants were encouraged to ask any questions regarding the questionnaire, which were addressed by the two researchers. After allowing sufficient time for completion, eight incomplete questionnaires exhibiting consistent or Z-type selection trends were excluded, resulting in a collection of 2026 valid questionnaires with an effective rate of 99.6% (2026/2034). The final sample consisted of 1916 males and 110 females aged between18-54 years (mean age:22.9 ± 3.7). All participants in this study belonged to various categories within the People's Liberation Army including active duty personnel (AD), military civilian staff members (MCT), and service persons employed by enterprises but stationed at troops (SPE). Their participation was voluntary, and they signed informed consent forms prior to inclusion in this study. Ethical approval for this study was obtained from the Ethics Committee of Strategic Support Force Medical Center (batch number: K2019 No.89).

### Questionnaire administration

We utilized a self-administered instrument for data collection, comprising primarily of the general demographic scale and the Chinese Maslach Burnout Inventory-General Survey (MBI-GS in Chinese).

The general demographic scale, an independently developed tool by researchers, encompasses variables such as gender, age, marital status, educational attainment, length of military service, military affiliation, working hours, and household income.

The Chinese Maslach Burnout Inventory-General Survey (MBI-GS), developed by Schanfeli & Maslach [39] and revised by Professor Li Chaoping & Shi Kan of the Chinese Academy of Sciences [40], is a validated tool for assessing burnout among Chinese individuals, and the scale has been published in the Acta Psychologica Sinica and is freely available for academic discussion and scientific research [41]. This scale utilizes a Likert 7-point measurement scale consisting of 15 items, ranging from "0 = never" to "6 = every day," to measure the intensity of burnout. The overall burnout score (OB) is calculated as the average score across all items, with higher scores indicating greater levels of burnout. The scale comprises three dimensions: emotional exhaustion (EE), depersonalization (DP), and professional achievement (PA). Scores for each dimension are obtained by averaging the scores for all items within that dimension, with PA being reverse scored. In this study, weighting coefficients were assigned to each factor to establish a corresponding score based on the original scale equation (OB = 0.4 EE + 0.3 DP + 0.3 PA). The total burnout score was then divided into three categories: low or non-burnout (0–1.49 points), medium level (1.50–3.49 points), and severe level (3.50–6 points) [42–44]. We employed the method for evaluation purposes while defining assessment at the dimensional level as total score evaluation.

### Data analysis

The data organization in this study was conducted by a single researcher, while the authenticity of the data was examined by two researchers. Statistical analysis was performed using SPSS 25.0 to accomplish the following tasks: (i) Normality tests were conducted on econometric data, with median (M) and quartiles (P_25_, P_75_) used to describe non-normal distributions, and mean and standard deviation $(\overline{x}\pm{S})$ used to describe the level of scores for each factor. (ii) For demographic characteristics related to burnout, Mann Whitney U test (Value for Z) was employed for comparing two groups, while Kruskal Wallis H test (Value for H) was utilized for comparing multiple groups.

## Results

The demographic and professional characteristics of the 2026 participants are presented in Table 1. A majority of the participants were male (95.3%), unmarried (67.3%), holding an Associate Degree (73.1%), below the age of 30 (87.9%), and had less than ten years of military service experience (88.7%). Furthermore, a significant proportion of their families reported an annual income below 100,000 RMB (64.1%). Upon analysis, it has been determined that gender, marital status, and identity of military do not have a significant influence on the score and dimensions of burnout. As a result, we have streamlined the relevant descriptions.

**Table 1:** Characteristics of the sample(*N* = 2026)

Variable	Subcategory	*N* (%)	Mean (*Standard Deviation*)
Age(years)	18–20	521(25.7)	22.9(3.7) years
	20–25	843(41.6)	
	25–30	417(20.6)	
	> 30	245(12.1)	
Educational level	High school diploma	689(34.0)	
	Associate degree	763(37.7)	
	Bachelor degree	477(23.5)	
	Master or PhD	97(4.8)	
Length of military service(years)	≤ 10	1797(88.7)	3.7 (4.6) years
	> 10	259(11.3)	
Average work hours per day(hours)	≤ 8 h	1506(74.3)	8.34(0.6)hours
	8 ~ 9 h	346(17.1)	
	≥ 9 h	174(8.6)	
Household income per year(RMB)	≤ 100,000	1298(64.1)	
	100,000 ~ ≤ 150,000	500(24.7)	
	150,000 ~ ≤ 200,000	111(5.5)	
	≥ 200,000	117(5.8)	

### The prevalence of burnout

The mean values for emotional exhaustion (EE), depersonalization (DP), personal achievement (PA), and overall burnout (OB) were 2.69 ± 0.89, 3.58 ± 0.92, 3.81 ± 0.85, and 3.37 ± 0.73. Following the aforementioned cutoff values, Table 2 presents the prevalence of burnout and its dimensions. A substantial proportion (97.5%) reported experiencing a medium or severe level of emotional exhaustion, while 93.6% reported a similar level of depersonalization. Furthermore, 97.3% experienced a sense of low professional accomplishment at a medium or severe level. In aggregate, 43.2% of participants reported severe burnout levels, with an additional 54.3% reporting medium levels.

**Table 2:** Detection situation of burnout (*N* = 2026)

Variable	Severe	Medium	Low or Non
Overall Burnout	875(43.2%)	1100(54.3%)	51(2.5%)
Emotional Exhaustion	355(17.5%)	1542((76.1%)	129(6.4%)
Depersonalization	1428(70.5%)	536(26.5%)	62(3.1%)
Professional Accomplishment	1629(80.4%)	343(16.9%)	54(2.7%)

### Demographic factors related to burnout in variable analysis

There were significant differences among age in years, educational level, length of military service in years, household income per year, and average work hours per day in hours. Table 3 summarizes different burnout among demographic characteristics of military personnel.

**Table 3:** Different burnout among demographic characteristics of military personnel(*N* = 2026)

Variable	Subcategory	Overall Burnout	Emotional Exhaustion	Depersonalization	Professional Accomplishment
Age(years)	18–20	3.33(3.20,3.67)	2.40(2.00,2.80)	3.75(3.00,4.00)	4.00(3.50,4.17)
	20–25	3.40(3.07,3.80)	2.60(2.20,3.20)	3.75(3.25,4.00)	4.00(3.67,4.33)
	25–30	3.40(3.20,3.80)	2.80(2.20,3.40)	3.75(3.50,4.00)	4.00(3.50,4.33)
	> 30	3.60(3.20,3.93)	2.60(2.20,3.45)	3.75(3.50,4.25)	4.00(3.67,4.33)
	H	36.41**	59.86**	21.46**	8.92*
Educational level	High school diploma	3.33(2.93,3.67)	2.40(2.00,3.00)	3.75(2.75,4.00)	3.83(3.33,4.17)
	Associate degree	3.40(3.07,3.80)	2.40(2.00,3.20)	3.75(3.25,4.00)	4.00(3.07,3.80)
	Bachelor degree	3.53(3.27,3.87)	2.80(2.40,3.60)	3.75(3.25,4.00)	4.00(3.67,4.33)
	Master or PhD	3.40(3.00,3.87)	2.60(2.20,3.30)	3.75(3.25,4.25)	3.83(3.33,4.16)
	H	58.50**	97.19**	36.29**	29.68**
Length of military service(years)	≤ 10	3.40(3.07,3.73)	2.60(2.00,3.20)	3.75(3.25,4.00)	4.00(3.50,4.33)
	> 10	3.60(3.13,4.12)	2.60(2.20,3.80)	4.00(3.25,4.50)	4.00(3.67,4.50)
	Z	-3.95**	-3.64**	-3.46**	-2.19*
Average work hours per day(hours)	≤ 8 h	3.40(3.07,3.67)	2.40(2.00,3.00)	3.75(3.25,4.00)	4.00(3.50,4.17)
	8 ~ 9 h	3.47(3.00,3.93)	2.60(2.00,3.40)	3.75(3.25,4.25)	4.00(3.67,4.33)
	≥ 9 h	3.93(3.33,4.80)	3.40(2.40,4.60)	4.13(3.75,5.00)	4.50(3.83,5.00)
	H	119.04**	103.46**	94.97**	119.04**
Household income per year(RMB)	≤ 100,000	3.40(3.13,3.87)	2.60(2.20,3.20)	3.75(3.25,4.00)	4.00(3.67,4.33)
	100,000 ~ ≤ 150,000	3.40(3.00,3.73)	2.60(2.00,3.15)	3.75(3.00,4.00)	4.00(3.38,4.17)
	150,000 ~ ≤ 200,000	3.27(2.27,3.80)	2.40(2.00,3.00)	3.50(2.00,4.00)	3.67(2.50,4.17)
	≥ 200,000	3.33(2.00,3.67)	2.20(2.00,3.20)	3.50(2.00,4.00)	3.83(2.00,4.00)
	H	36.05**	22.29**	33.17**	84.80**

**, < 0.01; *, *P* < 0.05; H Value for Kruskal Wallis H test, Z Value for Mann Whitney U test

### Multiple logistic regression analysis of burnout

Based on the results of multiple logistic regression analysis, utilizing the aforementioned five meaningful variables as independent variables and severe and moderate burnout as dependent variables, our findings indicate that age, working hours, length of military service, and household income significantly influence the detection of burnout (refer to Table 4).

**Table 4:** Multiple logistic regression analysis of burnout

Variable	*B*	*Wald (χ2)*	*OR*	*95%CI*	*P-Value*
Age(years)					
18–20	4.83	31.04	125.82	22.96,689.35	0.01
20–25	4.91	32.93	135.44	25.33,724.14	0.01
25–30	4.80	29.86	121.26	21.69,677.82	0.01
> 30	Reference				
Educational level					
High school diploma	0.81	1.74	2.24	0.68,7.41	0.18
Associate degree	0.63	1.06	1.89	0.56,6.32	0.30
Bachelor degree	0.28	0.20	1.32	0.38,4.50	0.66
Master or PhD	Reference				
Length of military service(years)					
≤ 10	-0.84	10.48	0.43	0.26,0.72	0.01
> 10	Reference				
Average work hours per day(hours)					
≤ 8 h	4.68	20.22	107.56	14.00,826.41	0.01
8 ~ 9 h	4.55	18.75	94.18	12.03,737.01	0.01
≥ 9 h	Reference				
Household income per year(RMB)					
≤ 100,000	-3.08	53.18	0.05	0.02,0.11	0.01
100,000 ~ ≤ 150,000	-4.06	72.42	0.02	0.01,0.04	0.01
150,000 ~ ≤ 200,000	-4.15	45.46	0.02	0.01,0.05	0.01
≥ 200,000	Reference				

*OR* Odd ratio, *CI* Confidence interval

## Discussion

Burnout is on the rise across various occupational populations worldwide [45]. In our research, we discovered that 875 respondents (43.2%) were identified as being at high risk for burnout, while 1100 (54.3%) reported moderate levels of burnout. Only 51 individuals (2.5%) indicated low or non-existent levels of burnout among the respondents. This finding highlights the significant work-related stress faced by military personnel in plateau regions. In comparison to a study conducted by Chinese academics [46], which revealed a serious prevalence of burnout among armed police officers with detection rates of mild, moderate, and severe burnout at 54.4%, 34.7%, and 2.2% respectively, it is evident that the level of burnout in military work within plateau regions is more severe. Previous research on different environments has consistently shown that military personnel experience significantly higher levels of burnout in special work settings compared to urban areas [47]. A single-center cross-sectional study conducted on military personnel stationed on the Xinjiang Plateau reported moderate and severe levels of burnout at rates of 22.03% and 7.91% respectively [48]. The potential reasons for this inconsistency with our study's results may be attributed to variations in geographical location and population selection criteria employed across studies. The inconsistency in the study results can be attributed to factors such as geographical location and population selection. Previous research has demonstrated that new geographic areas, weather conditions, high-injury combat situations, and other characteristics of modern warfare pose significant challenges to the mental well-being of military personnel [22, 49]. Consequently, burnout has emerged as a crucial aspect of mental health that contributes to decreased loyalty, undermines army morale, and hampers the enhancement of combat effectiveness [50].

In terms of demographic data, our study found no significant associations between gender, marital status, and identity of military with burnout. These findings align with previous studies [51–53], but contradict research indicating that women are more susceptible to burnout than men [54]. Furthermore, we observed a significant association between burnout and age among military personnel, consistent with Vojvodic's findings [19, 55]. Chappell's research in the United States Air Force also supports our results by demonstrating a close relationship between working hours and length of military service with burnout [56]. Additionally, excessive workload and long working hours can exacerbate burnout among military personnel [23, 57]. Moreover, we discovered a correlation between educational level and burnout similar to previous research finding [58]. As military personnel engage in diverse job content and responsibilities based on their educational experiences, higher education levels may lead to increased work-related stress.

It is noteworthy that in this study, we have identified a previously underexplored association between household income and burnout among military personnel, which has received limited attention in prior research within the military context. Notably, a study conducted on safety professionals reported a correlation between household income and personal accomplishment [59], while another study confirmed a relationship between household income and emotional well-being among medical students [60]. These findings should capture the attention of military managers due to the unique working environment and burdens experienced by individuals stationed in plateau regions, this is despite the fact that the household income in the survey is higher than the average household income in China. Financial compensation may potentially alter the perception of military personnel as they perceive their income to be commensurate with their labor contributions.

Our study was not originally designed to investigate the effects of smoking, but the prevalence of smoking among many military personnel makes the effects of smoking impossible to ignore [61]. On the one hand, smoking is a known risk factor for job-related outcomes at plateau regions [62, 63]. In addition, the impact of smoking on the cardiovascular and cerebrovascular system is significant, and can affect the endurance and physical strength of soldiers [64]. Smoking at plateau regions exacerbates the clinical condition of various diseases [65, 66]. While there are no consistent reports, some studies suggest a growing relationship between smoking and acute mountain sickness, a widespread condition that limits the ability to work at plateau regions, including at military sites [67]. However, a recent systematic review confirmed that there was no significant association between smoking and acute mountain sickness [68, 69], but acknowledged the risk of classification errors and high bias in studies examining the effects of smoking at plateau regions. However, given the high prevalence of smoking in military Settings and its harmful effects on many situations, it is theoretically plausible that there may be a link between smoking and burnout among military personnel. Therefore, further research on this topic is warranted.

### Limitations and strengths

Firstly, a major limitation of this study is the presence of measurement bias, which is subjective due to the researcher's selection of participants. Secondly, although this study is multicenter in nature, it only includes two provinces—Tibet and Qinghai. Therefore, we cannot generalize our findings to all military personnel in the plateau region and there may be a potential for selection bias. Lastly, given the cross-sectional design of our study, we are unable to conduct longitudinal analysis on the collected data. Furthermore, our study did not take into account potential factors that could impact burnout, such as environmental and physiological influences. But this serves as motivation for us to conduct more comprehensive follow-up research in the future.

However, it should be noted that our research utilizes appropriately validated tools that have been adapted to suit our specific context and possess strong psychometric properties for assessing primary outcomes. Furthermore, our study provides valuable insights into the prevalence of burnout among military personnel in the plateau region and identifies potential influencing factors. In light of these findings, addressing occupational health concerns among military personnel in this region becomes imperative.

## Conclusions

The present study provides empirical evidence that military personnel in plateau areas exhibit a relatively high prevalence of burnout, with 43.2% reporting severe occupational burnout. Specifically, factors such as working hours, age, military experience, and family income significantly contribute to this phenomenon. These findings underscore the necessity for special attention towards the occupational health of soldiers stationed in high-altitude regions to prevent burnout and mitigate other psychological issues. Moreover, these results offer valuable first-hand insights that can inform policy formulation and facilitate the implementation of effective intervention strategies aimed at enhancing career development among soldiers deployed in such challenging environments.

## Data Availability

The datasets used and analysed during the current study are available from the corresponding author on reasonable request with the permission of the Ethics Committee's relevant responsibilities.
